# Synthesis and Quasi-Static Compressive Properties of Mg-AZ91D-Al_2_O_3_ Syntactic Foams

**DOI:** 10.3390/ma8095292

**Published:** 2015-09-11

**Authors:** David B. Newsome, Benjamin F. Schultz, J.B. Ferguson, Pradeep K. Rohatgi

**Affiliations:** Materials Science and Engineering Department, University of Wisconsin-Milwaukee, 3200 N. Cramer St., Milwaukee, WI 53211, USA; E-Mails: dnewsome@uwm.edu (D.B.N.); jbf2@uwm.edu (J.B.F.); prohatgi@uwm.edu (P.K.R.)

**Keywords:** syntactic foam, metal matrix composite, energy absorption, compressive properties

## Abstract

Magnesium alloys have considerably lower density than the aluminum alloy matrices that are typically used in syntactic foams, allowing for greater specific energy absorption. Despite the potential advantages, few studies have reported the properties of magnesium alloy matrix syntactic foams. In this work, Al_2_O_3_ hollow particles of three different size ranges, 0.106–0.212 mm, 0.212–0.425 mm, and 0.425–0.500 mm were encapsulated in Mg-AZ91D by a sub-atmospheric pressure infiltration technique. It is shown that the peak strength, plateau strength and toughness of the foam increases with increasing hollow sphere wall thickness to diameter (*t*/*D*) ratio. Since *t*/*D* was found to increase with decreasing hollow sphere diameter, the foams produced with smaller spheres showed improved performance—specifically, higher energy absorption per unit weight. These foams show better performance than other metallic foams on a specific property basis.

## 1. Introduction

**Figure 1:**
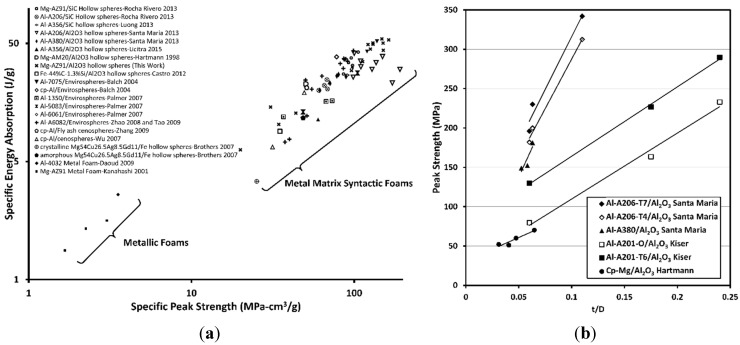
(**a**) Log–log plot of specific peak strength and specific energy absorption for different types of foams [2,4,5,6,7,8,9,10,11,12,13,14,15,16,17,18]; (**b**) Effect of reinforcement wall thickness to diameter ratio (*t/D*) on the peak strength of various aluminum alloy and commercially pure magnesium-hollow Al_2_O_3_ sphere metal matrix syntactic foams [2,5,10,19].

Metal matrix syntactic foams (MMSFs) are metal matrix composites containing a high volume fraction of hollow reinforcements (typically ~50%) giving the composite characteristics similar to a metal foam. The compressive stress–strain curves of metal foams exhibit an initial elastic region up to a plateau stress which is maintained until the material becomes fully dense (typically at ~50% strain) [1]. Consequently, metal foams can absorb a large amount of energy during compression before the stress rapidly increases. The shape of MMSF stress–strain curves differ slightly from the typical behavior of metal foams, in that in many cases, the initial elastic region ends in a peak stress, after which the stress drops and then gradually increases until densification (defined herein as the past-peak strain at which the stress returns to the initial peak stress) [2,3]. The energy absorbed before densification may be determined by integrating the stress–strain curve up to the densification strain. An Ashby style log–log chart showing the specific peak strength *vs.* the specific energy absorption for a variety of open-celled and syntactic foams in the open literature is shown in Figure 1a. To eliminate errors based on differing definition of material properties, the peak strength and energy absorption properties were determined directly from stress–strain plots provided in each publication. This figure shows the potential superiority of MMSFs over metal foams in terms of both specific peak strength and specific energy absorption capacity. 

Although magnesium alloys and their composites have gained popularity due to their light weight and attractive properties, comparatively few studies on magnesium based metal matrix syntactic foams have been published. One of the main difficulties in the development of magnesium based composites by liquid metallurgy processes is the reactivity of magnesium with the typical hollow reinforcements available (including SiO_2_, fly ash, Al_2_O_3_ *etc.*). These reactions can have a deleterious effect leading to the breakage and infiltration of the spheres. Weise *et al.*, used a cold chamber high pressure die casting technique to infiltrate a Mg alloy into preforms of 3M S60HS hollow glass bubbles (soda-lime-borosilicate glass) [20]. The combination of high infiltration pressure and the fracture of the spheres due to their reaction with the Mg alloy resulted in complete infiltration of the hollow spheres. 

Under certain processing conditions, the reaction between the magnesium and the reinforcement can be limited, or can even have a beneficial effect such as to improve bonding between the spheres and the matrix. Daoud *et al.*, synthesized Mg-ZC63- 25 vol% Fly Ash (61 wt % SiO_2_, 25 wt % Al_2_O_3_, 5 wt % Fe_2_O_3_, 1.6 wt % MgO and 1 wt % TiO_2_) composites via a stir casting method [21]. The magnesium alloy was shown to have reacted with the spheres to form a surface layer of MgO, which aided in wetting and incorporation of the spheres in the Mg-ZC63 matrix. Despite the reaction, the spheres remained largely intact and empty during the stir mixing and casting process. 

In a study by Hartmann *et al.*, magnesium based alloy-Al_2_O_3_ hollow sphere syntactic foams were successfully made by a sub-atmospheric pressure infiltration technique designed to limit the amount of contact between the magnesium alloy and the Al_2_O_3_ spheres, thus limiting reaction [10]. In their work, a pressure of 0.5–2.9 bar (typically 0.5 bar) was used to infiltrate commercially pure (cp)-Mg, Mg-AM20, Mg-AM50 and Mg-AZ91 into a preform of hollow sintered alumina spheres with *t/D* ratios varied between 0.03–0.06. Their work suggests that a threshold pressure exists beyond which, cracking and infiltration of the spheres occurs. Defouw *et al.*, used a pressure infiltration technique to synthesize pure Mg and Mg-AZ91-carbon microsphere syntactic foams [22]. In that study, the density of the foams was varied by adjusting the infiltration pressure from 200 to 800 kPa relative to vacuum. Foam densities from 0.7 to 1.03 g/cc were achieved with microstructures containing varied amounts of filled spheres and porosity due to lack of infiltration. Higher density foams were stronger, reaching peak strengths of 53 and 25 MPa for the AZ91 and pure Mg foams respectively. The overall energy absorption was 3–9 J/g independent of the density of the foam.

Brothers *et al.*, synthesized a low density, low melting point amorphous Mg_60_Cu_21_Ag_7_Gd_12_ alloy syntactic foam incorporating a network of crystalline iron hollow spheres by pressure infiltration [6]. Differential scanning calorimetry studies of the amorphous alloy and foam showed little to no deterioration of the glass-forming ability of the alloy as a result of the infiltration process. Selected specimens were annealed to crystallize the matrix and both amorphous and crystalline syntactic foams were tested in compression. Amorphous foams exhibited a significantly larger peak strength and stiffness compared to the crystalline foams. The incorporation of the ductile iron spheres significantly improved the compressive failure strain and energy absorption compared to the monolithic amorphous Mg alloy in compression. 

Recently, the quasistatic and high strain rate compressive mechanical properties of SiC hollow sphere reinforced Mg-AZ91 syntactic foams have been studied [4,23]. Rocha Rivero found that the incorporation of SiC hollow spheres (1 mm nominal diameter, 70 μm wall thickness) resulted in refinement of the microstructure surrounding the spheres in comparison to the unreinforced alloy. EDS of the matrix surrounding the spheres showed the presence of small amounts of Silicon, however this did not seem to have a significant effect on the intermetallics formed (chiefly Mg_17_Al_12_, β-phase) [4]. In both studies, Mg-AZ91-SiC syntactic foams exhibited high specific peak strengths and specific energy absorption under quasistatic conditions. Rocha Rivero found that under high strain rate conditions up to 726/s, the Mg-AZ91-SiC syntactic foams do not exhibit strain rate dependence suggesting that the strain rate sensitivity of these materials are governed by the properties of the matrix, which shows strain dependence only at higher strain rates (>1000/s) [4,24]. Further testing of these materials by Anantharaman *et al.*, at higher strain rates (1330–2300/s) did in fact show strain rate dependence, where peak strength increased with increasing strain rate [23].

One of the key attributes of the reinforcement that influences the properties of MMSFs is their wall thickness (*t*) to diameter (*D*) ratio. A cross-section of the published peak strength data for a variety of Al alloy and cp-Mg based MMSFs is plotted *versus* the *t/D* ratio of the reinforcement in Figure 1b, and shows a trend of increasing peak strength as the *t/D* ratio increases. The mechanical properties of the matrix are also known to influence the peak strength of MMSFs, as demonstrated in Figure 1b for aluminum alloys A201, A206 and A380. While Hartmann *et al.*, examined the effects of the hollow sphere *t/D* ratio (ranging from 0.03 to 0.06) on the peak strength of cp-Mg and several Mg alloys, they unfortunately only report the full range of data for the cp-Mg syntactic foam. However, their work suggests that the use of high strength magnesium alloys will have a similar complementary effect to the peak strength of syntactic foams as is the case for aluminum alloys. 

In the present study, high strength magnesium alloy AZ91D/Al_2_O_3_ syntactic foams with *t/D* ratios ranging from 0.06–0.1 are synthesized by a sub-atmospheric pressure infiltration technique, and the mechanical properties and sphere/matrix interface are investigated.

## 2. Results and Discussion

**Figure 2:**
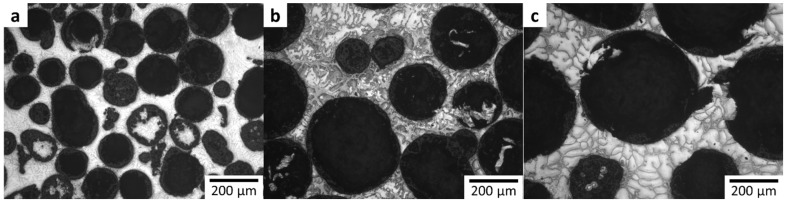
Representative microstructures of Mg AZ91D-Al_2_O_3_ syntactic foams containing hollow particles with diameters (**a**) 0.106–0.212 mm; (**b**) 0.212–0.425 mm and (**c**) 0.425–0.500 mm.

Representative micrographs of the as-cast composites are shown in Figure 2. The hollow particles appear to be uniformly distributed, and fully encapsulated by the metal matrix with little to no visible porosity in the matrix between the neighboring hollow particles. 

As can be seen in Figure 2a, a portion of the spheres were broken and their interiors were infiltrated at this pressure. The bulk densities of the reinforcements in each size range are presented in Table 1. It is probable that a portion of the infiltrated spheres shown in Figure 2a had been fractured prior to processing, as the flotation technique employed for eliminating fractured spheres for the larger sphere sizes is not capable of separating spheres with a bulk density greater than 1.483 g/cm^3^ (the density of chloroform). 

**Table 1:** Measured hollow sphere density for differing size ranges.

Sieve Size Range (mm)	Hollow Sphere Bulk Density (g/cc)
0.106–0.212	2.03
0.212–0.425	1.33
0.425–0.85	1.24

The Al_2_O_3_ hollow particles used in this investigation were on average spherical, however, especially in the case of the finest reinforcement size, the shape of the exterior and interior walls exhibit some asymmetry. The thickness of the wall therefore can vary for each hollow particle. For these reasons (along with the difficulty in sectioning and polishing the spheres along the equatorial plane), quantifying the *t*/*D* ratio by traditional imaging techniques can be difficult. One method to determine the *t*/*D* ratio has been derived by considering the sphere geometry and the average density of the sphere, ρ*_sphere_*, as shown in Equation (1) where ${\text{ρ}}_{Al_{2}O_{3}}$ is the density of Al_2_O_3_ and *d* is the internal diameter of the hollow sphere [5].

(1) $$\rho_{sphere}=\rho_{Al_{2}O_{3}}\frac{\left(\frac{4}{3}\right)\pi \frac{D^{3}}{8}-\left(\frac{4}{3}\right)\pi \frac{d^{3}}{8}}{\left(\frac{4}{3}\right)\pi \frac{D^{3}}{8}}=\rho_{Al_{2}O_{3}}\left(1-\frac{d^{3}}{D^{3}}\right)$$

The *t*/*D* ratio of the hollow particles may then be determined from the densities of Al_2_O_3_ and hollow particles by Equation (2).

(2) $$\frac{t}{D}=\frac{1}{2}\left[1-{\left(1-\frac{{\text{ρ}}_{sphere}}{\rho_{Al_{2}O_{3}}}\right)}^{\frac{1}{3}}\right]$$

**Figure 3:**
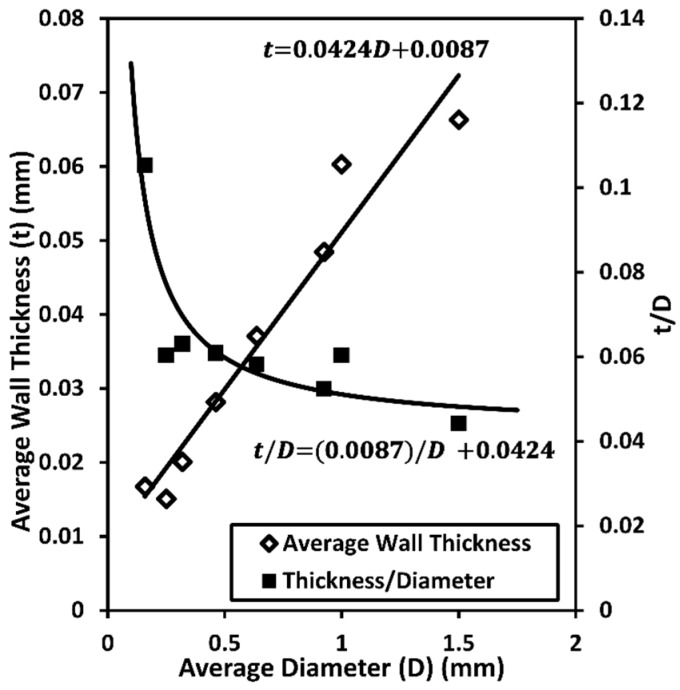
Dependence of average wall thickness and *t*/*D* ratio on reinforcement diameter.

Figure 3 shows that though the average hollow sphere wall thickness increases with increasing sphere diameter, the *t*/*D* ratio decreases asymptotically.

**Figure 4:**
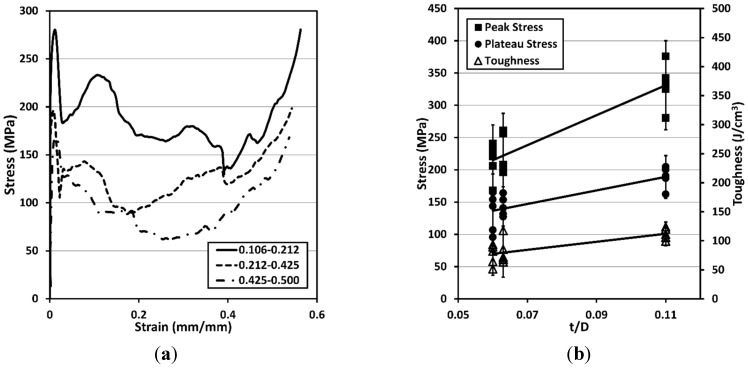
(**a**) Typical compressive stress–strain curves for Mg AZ91D-Al_2_O_3_ syntactic foams; (**b**) Peak strength (filled diamonds), plateau strength (filled squares) and toughness (open triangles) *vs.* *t*/*D* ratio. All properties shown increase with increasing *t*/*D* ratio. Error bars are shown (two standard deviations) based on the average value of each property.

Representative quasi-static engineering stress–strain curves are shown in Figure 4 for each size range. Each curve is typical of the quasistatic compression of MMSFs, in that an initial peak is reached (peak stress), followed by a sharp drop, and subsequent densification/shearing events that correspond to the shear of the MMSF at the critical shear stress, and crushing and densification of the hollow particles [5]. The smallest size reinforcements (0.106–0.212 mm) exhibited a significantly higher strength than the larger size reinforcements, though the densification strain (the past-peak strain at which the stress returns to the initial peak stress value) was roughly the same for each size range.

The measured peak strength, plateau strength, densification strain and energy absorption are reported in Table 2. The energy absorption (toughness) of the MMSFs was determined by calculating the area under the stress–strain curve up to the densification strain (the strain at which the stress reached the magnitude of the initial peak stress and subsequently continues to rise). The plateau strength reported in this work is the average of all of the measured stress data points between the strain corresponding to the initial peak and the densification strain. The specific energy absorption and specific peak strength of the MMSFs produced in this study are plotted on Figure 1a (shown with an “*x*” marker) for the purpose of comparison with metal foams of different compositions produced in other studies. The MMSFs containing the smallest Al_2_O_3_ hollow spheres, (0.106–0.212 mm) exhibited the best combination of specific peak strength and specific energy absorption, outperforming a variety of MMSFs and metal foams produced in other studies [2,4,5,6,7,8,9,10,11,12,13,14,15,16,17,18]. 

**Figure 5:**
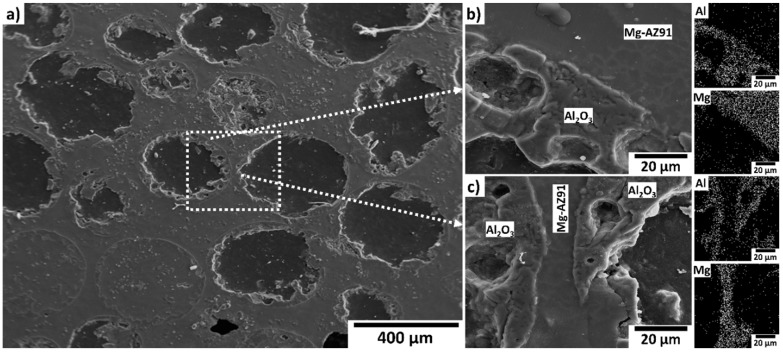
Scanning electron microscope (SEM) Micrographs and energy dispersive X-ray spectroscopy (EDS) dot maps of Mg-AZ91D/Al_2_O_3_ (0.425–0.5 mm) syntactic foam; (**a**) polished cross-section showing filled and empty hollow particles; (**b**) upper portion of left side sphere (within dashed box in Figure 5a) showing physical and mechanical bonding at the Mg/Al_2_O_3_ interface; (**c**) region between spheres (within dashed box in Figure 5a) showing physical and mechanical bonding at the Mg/Al_2_O_3_ interface.

Figure 4b shows a plot of each measured property in relation to the *t/D* ratio. Each measured property was observed to increase with increasing *t*/*D* ratio. Also of note, the peak strength of the MMSFs exceeds the yield strength of the matrix alloy Mg-AZ91D (160 MPa) [25]. This indicates that there is load transfer from the matrix to the reinforcement as a result of good bonding between the Mg-AZ91D alloy and the Al_2_O_3_ spheres. The peak stress in this case corresponds to the concurrent permanent deformation of the matrix and spheres at the critical shear stress. After this point, the compressive stress–strain curves for the MMSFs become non-linear, corresponding to plastic deformation of the matrix and further fracture of the spheres [5,26]. Closer examination of the interface between the Al_2_O_3_ spheres and the matrix via scanning electron microscope (SEM) (Topcon, Hasunuma-cho, Itabashi-Ku, Tokyo, Japan) in Figure 5 reveals that in many cases, the magnesium alloy has infiltrated pores in the walls of the hollow particles. The results of energy dispersive X-ray spectroscopy (EDS) (Advanced Analysis Technologies, Dane, WI, USA) dot mapping of aluminum (Al) and magnesium (Mg) in the magnified regions of Figure 5b,c are shown in the insets to the right of each image respectively. A good interface is observed between the surfaces of the spheres and the surrounding matrix suggesting that bonding has occurred. This combination of mechanical and chemical bonding between the matrix and the hollow particles is therefore expected to be responsible for the improvement in strength observed in these MMSFs over that of the base alloy.

**Table 2:** Summary of quasi-static compression data for Mg-AZ91D/Al_2_O_3_ syntactic foams.

Hollow Sphere Size Range (mm)	Peak Stress (MPa)	Plateau Stress (MPa)	Toughness (J/cm^3^)	Densification Strain (%)	Density (g/cm^3^)
0.106–0.212	342	162	99	60%	2.27
	325	200	120	59%	2.20
	280	187	106	56%	2.15
	376	205	124	59%	2.31
	332	190	111	57%	2.21
0.212–0.425	261	132	85	61%	1.59
	208	141	68	48%	1.90
	199	153	118	58%	1.98
	256	164	72	44%	1.91
	196	127	64	54%	2.10
0.425–0.500	241	165	93	56%	1.85
	221	154	89	58%	1.82
	230	144	82	56%	1.83
	168	96	52	54%	1.75
	206	107	64	58%	1.83

## 3. Experimental Section

Syntactic foams composed of Mg alloy AZ91D (Magnesium Elektron, West Des Moines, IA, USA) incorporating approximately 50 vol% hollow Al_2_O_3_ hollow particles (ALODUR white bubble alumina) (C-E Minerals Processing Inc., Newell, WV, USA) were synthesized. The nominal compositions of the alloy and the hollow particles are presented in Table 3 (information provided by suppliers). The hollow particles were supplied by C-E Minerals (C-E Minerals Processing Inc., Newell, WV, USA) in standard size (diameter) ranges of 0–0.5, 1–3 and 1–5 mm. The spheres were further sorted by size using an Allen Bradley Sonic Sifter (Allen Bradley Co., Milwaukee, WI, USA) to produce several size ranges although only 0.106–0.212, 0.212–0.425 and 0.425–0.500 mm were used to synthesize syntactic foams. After sorting, the Al_2_O_3_ particles were floated in chloroform (CHCl_3_) (Thermo Fisher Scientific, Waltham, MA, USA) which has a density of 1.483 g/cm^3^, causing the defective spheres to sink and the intact spheres to float, thus allowing them to be collected. The spheres with size range 0.106–0.212 were not floated in chloroform due to their higher bulk density. The bulk density of the hollow particles for each size range was determined by measuring the mass of a fixed amount of spheres and the corresponding volume of water displaced by these same spheres. The size ranges evaluated in this study along with their measured densities are shown in Table 1. 

**Table 3:** Nominal composition of matrix and reinforcements.

Material	Component	Nominal Content (wt %)
**AZ91D**	Mg	88–91
	Al	8.3–9.7
	Mn	0.13 min
	Zn	0.35–1.0
	Si	0.50 max
	Cu	0.1 max
	Ni	0.03 max
**Al_2_O_3_ Hollow Sphere**	Al_2_O_3_	98.8
	SiO_2_	0.8
	Na_2_O	0.1
	MgO	0.05
	Fe_2_O_3_	0.03
	CaO	0.03

A sub-atmospheric pressure infiltration apparatus was used to produce the MMSFs in this work. A 13.5 mm × 13.5 mm steel tube with one end welded shut was tap-packed with hollow particles to a height of 70–90 mm. An ingot of AZ91D alloy was placed above the hollow spheres, separated by a 2 mm thick layer of zirconia felt (Zircar Zirconia Inc., Florida, NY, USA). The felt was used as a reaction barrier between the magnesium alloy melt and the spheres prior to infiltration and served as a filter to remove the oxide layer from the liquid melt. The crucible containing the preform and ingot was heated in a quartz chamber under vacuum to 650 °C and held for 1 h at which time the alloy had fully melted and uniformly sealed the inner perimeter of the crucible. Argon gas (Airgas, Radnor Township, PA, USA) was then rapidly introduced into the heated quartz chamber, reaching a sub-atmospheric pressure of 0.4 bar within 10 s, thereby forcing the molten alloy into the evacuated spaces between the hollow particles. The quartz chamber containing the sample was then removed from the tube furnace and the steel crucible quenched immediately in room temperature water. The density of the composites was measured using a Mettler Toledo AT261 Delta Range Microbalance (Mettler Toledo, Columbus, OH, USA) equipped with a density measurement apparatus (Archimedes method) (Mettler Toledo, Columbus, OH, USA). The specimens were first lightly coated with vacuum grease to prevent infiltration of surface pores during the density measurement. Microstructural analysis was performed with a Nikon Eclipse TS100 microscope (Nikon, Brighton, MI, USA) equipped with an automated stage and Clemex Professional Image analysis software (Clemex Technologies, Inc., Longueuil, PQ, Canada), and a Topcon SM300 scanning electron microscope (SEM) (Topcon, Hasunuma-cho, Itabashi-Ku, Tokyo, Japan) equipped with energy dispersive X-ray spectroscopy (EDS) (Advanced Analysis Technologies, Dane, WI, USA) was used to observe the interfaces between the AZ91D matrix and Al_2_O_3_ spheres. To facilitate imaging, the specimens were sputter coated with gold/palladium using a Denton Vacuum Desk II sputter coater (Denton Vacuum LLC, Moorestown, NJ, USA).

Quasi-static compression testing was performed in accordance with ASTM C365-94 (ASTM International, West Conshohocken, PA, USA) on specimens having dimensions of approximately 13 mm × 13 mm × 12 mm (thickness). Testing was carried out using a SATEC Model 50Ud Universal Testing Machine (Instron, Norwood, MA, USA) at constant crosshead speed with an initial strain rate of 10^−3^ s^−1^ and a self-leveling platen. Strains were calculated from the crosshead displacement, and were corrected for deflection of the load frame. The quasi-static compression curves typically exhibited an initial peak followed by a lower plateau stress and later densification. Compression was stopped when the stress slightly exceeded the magnitude of the initial peak stress. 

## 4. Conclusions 

The microstructure and quasi-static mechanical properties of Mg-AZ91D-Al_2_O_3_ hollow sphere syntactic foams have been characterized for foams of three different microsphere sizes, specifically 0.106–0.212, 0.212–0.425 and 0.425–0.5. The peak strength, plateau strength and toughness of the AZ91D-Al_2_O_3_ syntactic foams increase with increasing *t/D* ratio. Because *t/D* was found to increase with decreasing sphere diameter, the foams produced with finer hollow particles result in improved performance. Good bonding between the matrix and the reinforcement as observed by SEM suggests that the increased strength of the MMSFs relative to the base alloy yield strength is as a result of load transfer during compression.
